# Berry texture QTL and candidate gene analysis in grape (*Vitis vinifera* L.)

**DOI:** 10.1093/hr/uhad226

**Published:** 2023-11-08

**Authors:** Hong Lin, Li Ma, Qiuyu Guo, Cheng Liu, Yangming Hou, Zhendong Liu, Yuhui Zhao, Changyue Jiang, Xiuwu Guo, Yinshan Guo

**Affiliations:** College of Horticulture, Shenyang Agricultural University, 120 Dongling Road, Shenyang, 110866, China; College of Horticulture, Shenyang Agricultural University, 120 Dongling Road, Shenyang, 110866, China; College of Horticulture, Shenyang Agricultural University, 120 Dongling Road, Shenyang, 110866, China; College of Horticulture, Shenyang Agricultural University, 120 Dongling Road, Shenyang, 110866, China; College of Horticulture, Shenyang Agricultural University, 120 Dongling Road, Shenyang, 110866, China; College of Horticulture, Shenyang Agricultural University, 120 Dongling Road, Shenyang, 110866, China; College of Horticulture, Shenyang Agricultural University, 120 Dongling Road, Shenyang, 110866, China; College of Horticulture, Shenyang Agricultural University, 120 Dongling Road, Shenyang, 110866, China; College of Horticulture, Shenyang Agricultural University, 120 Dongling Road, Shenyang, 110866, China; College of Horticulture, Shenyang Agricultural University, 120 Dongling Road, Shenyang, 110866, China; Ministry of Education Key Laboratory of Protected Horticulture, Shenyang Agricultural University, Shenyang, 110866, China

## Abstract

Berry texture is a noteworthy economic trait for grape; however, the genetic bases and the complex gene expression and regulatory mechanism for the diverse changes in berry texture are still poorly understood. In this study, the results suggest that it is difficult to obtain high-mesocarp firmness (MesF) and high-pericarp puncture hardness (PPH) grape cultivars with high pericarp brittleness (PerB). The high-density linkage map was constructed using whole-genome resequencing based on 151 F_1_ individuals originating from intraspecific hybridization between the firm-flesh cultivar ‘Red Globe’ and soft-flesh cultivar ‘Muscat Hamburg’. The total length of the consensus map was 1613.17 cM, with a mean genetic distance between adjacent bin markers of 0.59 cM. Twenty-seven quantitative trait loci (QTLs) for berry MesF, PPH, and PerB were identified in linkage groups (LGs) 1, 3, 4, 6, 8, 9, 10, 11, 14, 16, and 17, including twelve QTLs that were firstly detected in LGs 6, 11, and 14. Fourteen promising candidate genes were identified from the stable QTL regions in LGs 10, 11, 14, and 17. In particular, *VvWARK2* and *VvWARK8* refer to chromosome 17 and are two promising candidate genes for MesF and PPH, as the *VvWARK8* gene may increase pectin residue binding with WARK for high berry firmness maintenance and the allele for *VvWARK2* carrying the ‘CC’ and ‘GA’ genotypes at Chr17:1836764 and Chr17:1836770 may be associated with non-hard texture grape cultivars. In addition, real-time quantitative polymerase chain reaction (RT–qPCR) verification revealed that the promising candidate transcription factor genes *VvMYB4-like*, *VvERF113*, *VvWRKY31*, *VvWRKY1*, and *VvNAC83* may regulate cell wall metabolism candidate gene expression for grape berry texture changes.

## Introduction

Grape (*Vitis vinifera* L.), which belongs to family Vitaceae, is an economically important fruit tree cultivated worldwide. Grape berry texture has high agronomic relevance because of its relationships with the quality parameters and marketing requirements of table, raisin, and wine grapes [1], and the flesh firmness and peel sensory characteristics are suggested to differentiate commercial grape cultivars [2]. A firm mesocarp (flesh) contributes to grape freshness and to a desirable crunchy texture, which is one of the most relevant characters in the breeding of table grape cultivars [3, 4]. It was found that berries of the hard cultivar ‘Red Globe’ were more firm, springy, and resilient but less hard and gummy than those of the hard cultivar ‘Crimson Seedless’ at the same ripening stage because the berries of ‘Red Globe’ have thicker skin than those of ‘Crimson Seedless’ [5], and the ‘Muscat Hamburg’ has been found to be a typical soft cultivar with only higher firmness than ‘Moscatuel’ [6]. In addition, a previous study revealed that *vinifera* wine and dual-purpose (wine and table) cultivars, such as ‘Chardonnay’ and ‘Terbash’, had soft and non-crisp flesh, and the *labruscana* grapes, which included table-use and dual-purpose cultivars, lacked crisp flesh texture cultivars due to *labruscana* cultivars generally having high DFP (the small deformation at the first major peak in flesh puncture test), which included table-use and dual-purpose cultivars [7]. These studies indicated that flesh firmness and skin thickness together affect berry texture.

Grape is a perennial woody liana with a complex genetic background and long growth cycle. Previous studies have found that the broad-sense heritability (*H^2^*) of berry firmness had a range of 0.813 to 0.94843 [8, 9], and increasing attention has been given to improving grape berry texture through genetic improvement [10, 11]. Nevertheless, the mechanism underlying berry texture differences between varieties is still poorly understood, especially the genetic basis between the flesh and peel. Quantitative trait locus (QTL) mapping and marker-assisted selection (MAS) based on a double pseudo-testcross strategy can significantly accelerate the breeding process and make grape breeding more precise and efficient [12, 13]. Carreño *et al.* [14] scored the firmness using the force (N) required to achieve a 20% deformation of the berry height, and found QTLs related to berry firmness in linkage groups (LGs) 1, 4, 5, 9, 10, 13, and 18, with these QTLs explaining up to 19.8% of the total phenotypic variance. In another follow-up study, they identified additional stable QTLs between the markers UDV125 and VMCNG2H2 in LG 8 and between the markers VVIN16 and VVCS1E103N17FM1 in LG 18 that explained 27.6% of the phenotypic variance, which was the first report of a QTL for grape berry firmness that was stable in different seasons [9]. Ban *et al.* [8] found two stable QTLs associated with berry firmness near SSR maker VMC2E7 in LG 3 and near SSR makers UDV073 and VVIH01 in LG 10 by using 98 individuals of the F_1_ population from 2016. In addition, further studies have detected multiple QTLs for berry firmness in LGs 2, 8, 15, 16, 17, and 18 using different grape crossing populations in recent years and inferred that the candidate genes *abscisic-aldehyde oxidase-like*, *endoglucanase 3*, *senescence-associated protein din1*, *expansin-A6*, *polygalacturonase*, *pectate lyase 4*, and *VviAGL11* are associated with berry firmness [10, 15–18, 11]. However, the QTLs for pericarp brittleness (PerB) remain largely unknown, and more studies need to explore the reason for berry texture differences between different cultivars.

Many previous studies have shown that the cell wall is one of the important factors that determines fruit firmness [19, 20, 21] and the berry texture changes are caused by the interaction of various cell wall hydrolases and regulatory proteins, due to the fact that the fruit cell wall is a dynamic network structure [19]. During fruit ripening and softening, the expression of genes related to cell wall-degrading enzymes leads to fruit firmness changes through pectin dissolution, neutral sugar loss, xylan depolymerization, and cell wall relaxation [20, 22–24], and genes coding for expansin (EXP), β-galactosidases (β-GALs), endo-1,4-β-glucanases, pectin methyl-esterases (PMEs), pectate lyases (PLs), xyloglucan endo-transglycosylases (XTHs), and polygalacturonases (PGs) are usually associated with fruit firmness [20, 22–29]. In general, the grape berry texture in the young stage is relatively firm and rapidly decreases in firmness after veraison (onset of ripening), the crucial stage of berry softening [30, 31], and berry firmness is defined by the interaction of numerous genes and pathways associated with degradation of the cell wall and cuticle properties [9]. In addition, a large number of studies have found that the transcription factors (TFs) such as the NAC, MADS-box, ERF, and bHLH families can regulate fruit softening by changing fruit texture [32, 33, 34, 35]. The NAC TF NOR-like1 has been found that positively regulate the fruit softening by changing the expressions of *SlPG2a*, *SlPL*, *SlCEL2* and *SlEXP1* [36], and overexpressed or repressed the expression of *SlNAC1* displayed earlier or delayed softening [37, 34], and the NAC-MYB module has been proven to regulate secondary cell wall biosynthesis in peach fruit [38]. Moreover, the SlERF.F12 was shown to negatively regulate the fruit softening by repressing the expression of cell wall genes *SlPG2a* and *SlPL* [39]. Nevertheless, how TFs regulate gene expression to influence berry texture in grapes remains to be determined.

In this study, to better understand the genetic determinants of and key genes for berry texture in grapes, intraspecific hybridization was performed between ‘Red Globe’ and ‘Muscat Hamburg’, which show significant differences in the main berry texture traits (MesF, PPH, and PerB). The main berry texture trait-related QTLs were detected based on a high-density whole-genome resequencing genetic map from 2017 to 2019, and promising candidate genes were analysed based on the 12X.v2 grapevine genome in the URGI database.

## Results

### Berry texture analysis

**Figure 1:**
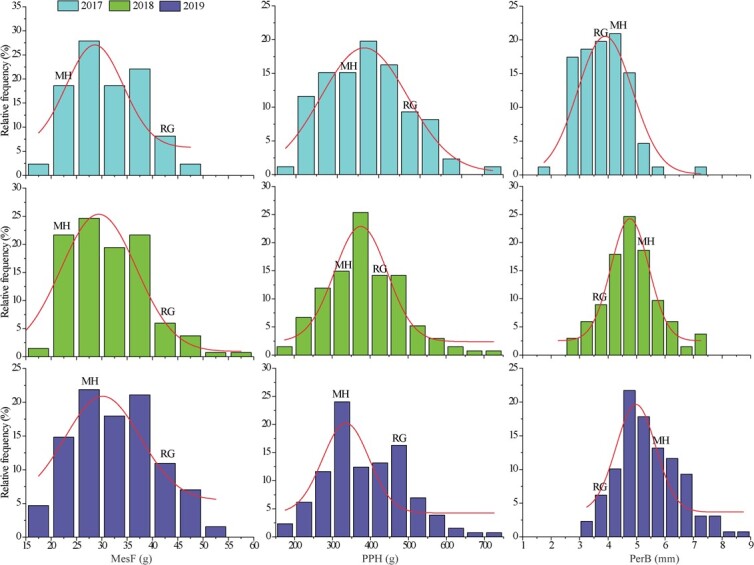
Distribution of MesF, PPH, and PerB in the ‘Red Globe’ × ‘Muscat Hamburg’ population. Histograms show data variations from 2017 to 2019. RG and MH indicate female parent ‘Red Globe’ and male parent ‘Muscat Hamburg’ mean values, respectively.

In this study, grape berry MesF, PPH, and PerB exhibited continuous variation and a normal distribution in the ‘Red Globe’ × ‘Muscat Hamburg’ (RM) population from 2017 to 2019, and most of the three berry texture traits in RM showed wide transgressive segregation (Fig. 1). The mean values of MesF, PPH, and PerB in offspring were 31.23–32.64 g, 372.54–385.36 g, and 3.89–5.41 mm, respectively, from 2017 to 2019, which were always between those of female ‘Red Globe’ and male ‘Muscat Hamburg’. The broad sense heritability (*H^2^*) for PerB, PPH, and MesF was 0.89–0.96 in RM from 2017 to 2019, especially for PPH, which showed a large and stable *H^2^* (0.96) in the threee consecutive years (Table 1).

**Table 1:** Descriptive statistical parameters for berry texture in the ‘Red Globe’ × ‘Muscat Hamburg’ population.

Texture	MesF/g			PPH/g			PerB/mm		
	2017	2018	2019	2017	2018	2019	2017	2018	2019
Red Globe	43.66	44.32	42.87	431.61	429.26	471.2	3.57	3.48	3.68
Muscat Hamburg	23.44	23.96	25.12	302.45	318.93	317.79	4.4	5.13	5.74
Maximum	48.22	57.68	51.31	689.88	731.94	706.81	7.31	7.41	8.69
Minimum	16.41	15.29	15.84	188.33	192.4	177.33	1.74	2.54	3.09
Average	31.23	31.32	32.64	372.54	384.51	385.36	3.89	4.8	5.41
Heritability	0.89	0.91	0.93	0.96	0.96	0.96	0.87	0.88	0.91

**Figure 2:**
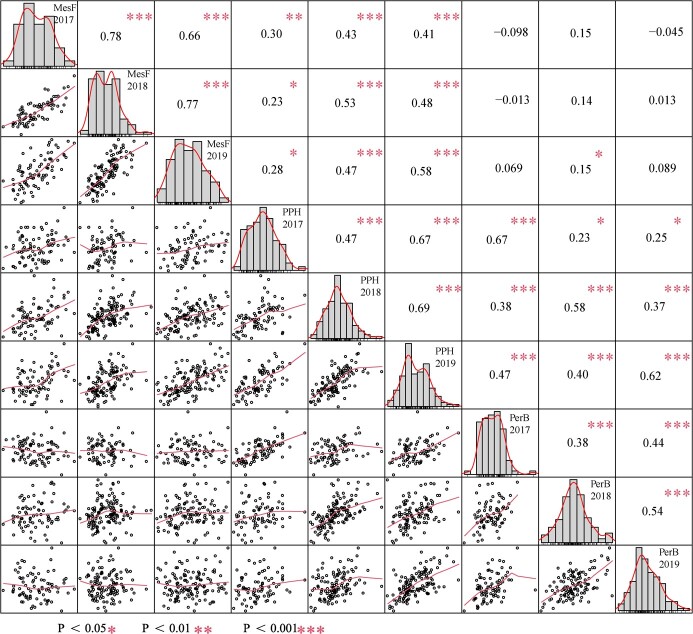
Phenotypic correlations between MesF, PPH, and PerB from 2017 to 2019. Numbers in the upper right corner represent strong or weak correlations, and the red asterisks indicate a significant difference.

Phenotypic correlations between berry texture traits (MesF, PPH, and PerB) averaged over the 3 years varied from −0.013 to 0.78 (Fig. 2). Each berry texture trait showed strong, significant positive correlations (*P* < 0.05) from 2017 to 2019, especially MesF, which showed the strongest positive correlations (0.66–0.78) in all three years. MesF and PPH showed a weak but highly significant positive correlation (0.23–0.58, *P* < 0.05), and PPH showed a strong significant positive correlation with PerB (0.23–0.67, *P* < 0.05). However, there was no significant correlation (*P* < 0.05) between MesF and PerB in this study.

### Quality evaluation of whole-genome resequencing

After filtering out raw reads, low-quality sequences, redundant reads and unpaired reads, whole-genome resequencing yielded 546.45 Gb of clean data, of which 18.18 Gb was from the female parent (‘Red Globe’), 19.58 Gb from the male parent (‘Muscat Hamburg’) and 508.69 Gb from the offspring. The Q30 and GC contents were calculated to be 92.77% and 35.90% for the female parent, 92.57% and 35.77% for the male parent, and 91.95% and 35.94% for the offspring, respectively (Table S1, see online supplementary material). The clean reads were then mapped to the reference genome (*V. vinifera* 12X.v2) using BWA software. A total of 96.64% of the clean reads mapped to the reference genome from the female parent, 96.44% from the male parent and 95.28% from the offspring. The average sequencing depth was 30× for the male parent ‘Muscat Hamburg’, 31× for the female parent ‘Red Globe’ and 5.07× for the offspring (Table S1, see online supplementary material).

### Identification of SNPs and InDels

**Figure 3:**
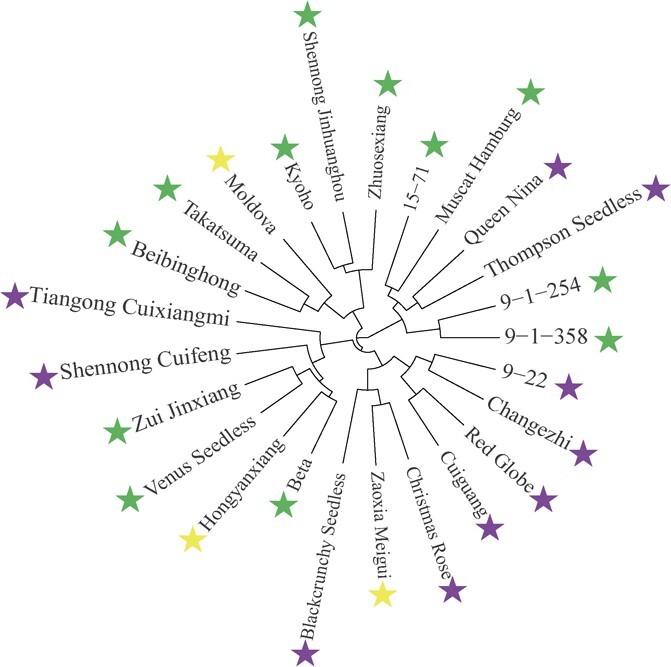
The genetic diversity of 25 grape cultivars (lines) with different berry textures. Purple, yellow, and green stars represent hard-, medium-, and soft-textured grape cultivars (lines), respectively.

A total of 3 224 217 single nucleotide polymorphisms (SNPs) and 597 344 insertions/deletions (InDels) were identified between ‘Red Globe’ and ‘Muscat Hamburg’, of which 1 278 507 and 681 862 SNPs were detected in intergenic regions and introns, respectively, and 66 854 SNPs were nonsynonymous. In addition, 1585 InDels resulted in codon deletion and insertion, 800 InDels resulted in codon changes (Tables S2 and S3, see online supplementary material), and the detected SNPs and InDels covered 19 chromosomes of the grape genome (Fig. S1, see online supplementary material). After filtering, 189 345 markers were retained, and these were classified into four different genotypes and used for linkage map construction. Approximately 19 684 of these markers were from the ‘unknown’ chromosomes (Table S4, see online supplementary material). To fully understand the genetic diversity and relatedness between ‘Red Globe’ and ‘Muscat Hamburg’, 25 grape cultivars (lines) with different berry textures were divided into three main groups by pedigree analysis using fifty-four 40–45 bp InDel markers randomly selected from 19 chromosomes of ‘Red Globe’ and ‘Muscat Hamburg’ and five KASP primers associated to SNPs in the gene or promoter area of the promising candidate genes for berry texture. The soft berry cultivar ‘Muscat Hamburg’ and hard berry cultivar ‘Red Globe’ were divided into different branches, and the soft berry and hard berry cultivars (lines) showed clear aggregation, especially six hard-cultivars were well clustered together, except for one medium-cultivars variety ‘Zaoxia meigui’ (Fig. 3).

### Genetic linkage map construction

A total of 93 127 SNPs were assigned to 2725 bin markers for the RM population (Table S5, see online supplementary material). The bin markers were divided into 19 LGs (Table 2; Fig. S2, Table S5, see online supplementary material), spanning a total genetic distance of 1613.17 cM. The genetic length of the LGs ranged from 68.64 to 103.10 cM, with an average genetic distance of 84.90 cM. The largest group was LG 16, and the smallest was LG 18 (Table 2).

**Table 2:** Main characteristics of linkage groups in ‘Red Globe’ × ‘Muscat Hamburg’ consensus maps.

Linkage group	Bin marker number	SNP number	Total distance (cM)	Average distance (cM)	Maximum gap (cM)	Gaps <5 cM (%)
LG 1	141	4557	89.53	0.63	7.49	97.86
LG 2	165	6054	83.90	0.51	4.14	100.00
LG 3	148	7463	78.22	0.53	9.45	99.32
LG 4	159	5902	82.38	0.52	8.26	99.37
LG 5	133	4465	94.09	0.71	17.22	96.97
LG 6	165	6934	78.58	0.48	5.23	99.39
LG 7	122	2574	72.23	0.59	3.07	100.00
LG 8	149	5087	91.67	0.62	16.30	98.65
LG 9	143	3328	87.70	0.61	9.85	97.18
LG 10	152	7370	70.76	0.47	3.78	100.00
LG 11	140	4605	85.97	0.61	9.45	99.28
LG 12	125	2663	88.00	0.70	5.23	99.19
LG 13	139	4142	100.88	0.73	14.50	99.28
LG 14	131	4248	92.12	0.70	15.84	98.46
LG 15	122	7508	77.16	0.63	7.53	96.69
LG 16	139	3028	103.10	0.74	16.30	98.55
LG 17	117	2440	72.16	0.62	13.62	98.28
LG 18	165	3964	68.64	0.42	3.07	100.00
LG 19	170	6795	96.08	0.57	14.94	98.82
Total	2725	93 127	1613.17			
Average	143.42	4901	84.90	0.59	9.75	98.80%

The number of SNP markers in the LGs ranged from 2440 to 7508. LG 15 and LG 17 contained the maximum and minimum number of SNP markers, respectively. The number of bin markers in the LGs ranged from 117 to 170. LG 17 and LG 19 contained the smallest and maximum number of bin markers, respectively (Table 2). Most of the LGs contained gap regions <5 cM, but there were no gaps >5 cM in LGs 2, 7, 9, 10, and 18. The maximum gap was 17.22 cM in LG 5 (Table 2). The mean genetic distance value of adjacent bin markers was 0.59 cM, ranging from 0.42 to 0.74 cM among the 19 LGs (Table S6, see online supplementary material). In this study, the marker order in the linkage map showed better collinearity with the physical map, and the Spearman correlation coefficients were higher than 0.98 for most LGs, which indicates high accuracy of genetic recombination rates (Fig. S3, Table S7, see online supplementary material).

### Identification of QTLs for berry texture

**Figure 4:**
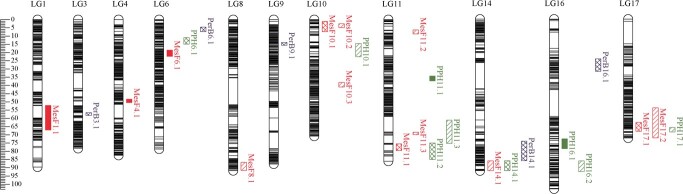
Stable QTLs for MesF, PPH, and PerB on the consensus map. Linkage group distances are in Kosambi cM. QTLs are shown on the right side and are named starting with the abbreviation of the trait (MesF for mesocarp firmness, PPH for pericarp puncture hardness, PerB for pericarp brittleness), followed by the linkage group number and the position of the identified QTL per trait. The QTLs with red, green, and blue colors are associated with MesF, PPH, and PerB, respectively. The QTLs with different solid lines, cross-hatching and vertical lines are QTLs identified in 2017, 2018, and 2019, respectively. Height of the QTL boxes indicates the confidence interval of QTLs.

According to the high-density bin-based genetic map, a total of 27 QTLs for MesF, PPH, and PerB were identified using multiple QTL mapping from 2017 to 2019 in the RM population (Fig. 4, Table 3), which were distributed in LGs 1, 3, 4, 6, 8, 9, 10, 11, 14, 16, and 17, as described below.

Thirteen QTLs for MesF were detected in LGs 1, 4, 6, 8, 10, 11, 14, and 17, explaining 5.7% to 20.7% of the phenotypic variation (*R^2^*); this includes the major QTL MesF10.2 detected in LG 10, with a logarithm of odds (LOD) value of 14.2 in 2019, which explained up to 20.7% of the total variance. Nine QTLs for PPH identified in LGs 6, 10, 11, 14, and 17 explained up to 25.8% of the total variance, with the largest LOD value of 13.9 in 2017. In addition, five QTLs for PerB were identified in LGs 3, 6, 9, 14, and 16, which explained 8.3% to 17.4% of the phenotypic variation (Table 3).

In this study, the QTLs were considered stable if it was identified for more than 2 years or more than two traits in the same LG region, and four stable QTLs regions for berry texture were identified in LGs 10, 11, 14, and 17 (Fig. 4). Two QTLs (MesF10.1 and MesF10.2) for berry MesF were identified in LG 10, which explained 13.1% and 20.7% of the total variance in 2018 and 2019, respectively. Regarding LG 11, four stable QTLs (MesF11.1, MesF11.3, PPH11.2, and PPH11.3) for berry texture (MesF and PPH) were identified in the confidence interval peak 68.6–75.1 cM in 2018 and 2019, with LOD scores ranging from 3.6 to 6.8 and explaining 6.8% to 11.9% of the phenotypic variation. In 2018 and 2019, the stable QTL region in LG 14 contained three QTLs (MesF14.1, PPH14.1, and PerB14.1) for the three berry texture traits (MesF, PPH, and PerB), accounting for 10.1%, 9.9%, and 15.1% of the phenotypic variation, respectively (Fig. 4, Table 3).

An important stable QTL region for MesF and PPH was detected in LG 17 in 2018 and 2019, which included the QTLs MesF17.1, MesF17.2, and PPH17.1, showing good overlap. The major QTL MesF17.1 explained up to 16.1% of the total phenotypic variance, with a LOD score of 8.1, and PPH17.1 explained up to 15.6% of the total phenotypic variance, with a LOD score of 7.7 (Fig. 4, Table 3).

### Identification of candidate genes for berry texture

According to the bin markers (Block396 and Block406, Block724 and Block742, Block1781 and Block1770, Block2298, and Block2255) anchored to the 12X.v2 grape reference genome, a total of 412 candidate genes were identified in LGs 10, 11, 14, and 17 with stable QTL confidence intervals (Table S8, see online supplementary material). However, only 14 potential candidate genes were considered promising candidate genes based on their potential functional annotations and the available literature, including nine involved in berry softening behaviors, such as cell wall remodeling and cellulose and pectin degradation, and five TFs considered to participate in many molecular regulatory mechanisms. These promising candidate genes include wall-associated receptor kinase (WARK), β-glucosidase, glucan endo-1,3-β-glucosidase, β-1,3-galactosyltransferase, fasciclin-like arabinogalactan protein (FLA), WRKY31, ERF113, NAC83, MYB4-like, and WRKY1 TFs (Table 4).

**Figure 5:**
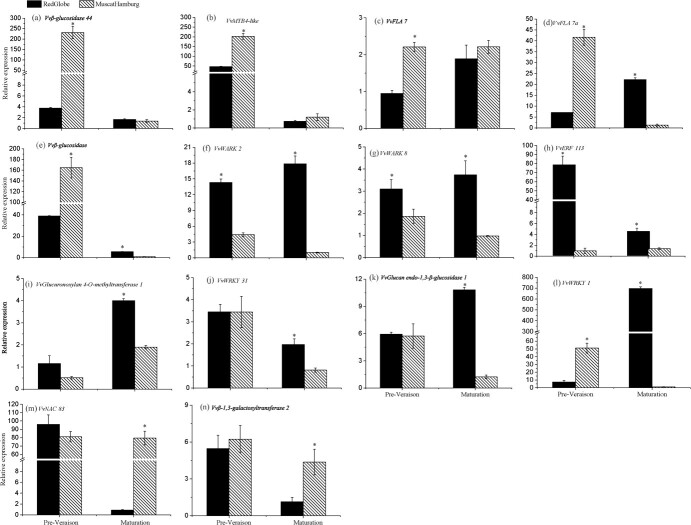
Expression of ten candidate genes in ‘Red Globe’ and ‘Muscat Hamburg’ at preveraison and maturation. Asterisks indicate significant differences detected by one-way ANOVA with Tukey’s test at *P* ≤ 0.05. Data are means ± SEs of three replications.

RT–qPCR validation showed that *Vvβ-glucosidase 44* (*Vitvi14g00996*) and *VvMYB4-like* (*Vitvi17g00231*) were downregulated in the male parent ‘Muscat Hamburg’ and female parent ‘Red Globe’ during ripening. The relative expression levels of *Vvβ-glucosidase 44*, *VvMYB4-like*, and *VvFLA 7* (*Vitvi11g00950*) were significantly higher in ‘Muscat Hamburg’ than in ‘Red Globe’ at preveraison, but the relative expression levels were not significantly different between ‘Red Globe’ and ‘Muscat Hamburg’ at maturation (Fig. 5a–c). The expression of the candidate gene *VvFLA7a* (*Vitvi11g00975*) and *Vvβ-glucosidase* (*Vitvi17g00234*) showed similar expression tendencies with *Vvβ-glucosidase 44* at preveraison but showed significantly higher in ‘Red Globe’ than in ‘Muscat Hamburg’ at maturation (Fig. 5d and e). The candidate genes *VvWARK2* (*Vitvi17g00175*) and *VvWARK8* (*Vitvi17g00209*) showed similar expression tendencies: the expression of both was higher in the female parent ‘Red Globe’ than in the male parent ‘Muscat Hamburg’ at preveraison and maturation, and expression in ‘Muscat Hamburg’ was downregulated during ripening (Fig. 5f and g). *VvERF113* (*Vitvi17g00025*) showed an expression tendency similar to those of *VvWARK2* and *VvWARK8* during ripening, but *VvERF113* was downregulated in ‘Red Globe’ from preveraison to maturation (Fig. 5h). At preveraison, the expression of the candidate gene *VvGlucuronoxylan 4-O-methyltransferase 1* (*Vitvi17g00106*) showed no significant difference between ‘Muscat Hamburg’ and ‘Red Globe’ but was higher in ‘Red Globe’ than in ‘Muscat Hamburg’ at maturation (Fig. 5i). Expression of the TF *VvWRKY31* (*Vitvi10g00063*) was higher in ‘Red Globe’ than in ‘Muscat Hamburg’ at maturation, although there was no difference at preveraison (Fig. 5j). *VvGlucan endo-1,3-β-glucosidase 1* (*Vitvi10g00085*) showed an expression tendency similar to *VvWRKY31*, except for being up-regulated in ‘Red Globe’ at maturation (Fig. 5k). The expression of *VvWRKY1* (*Vitvi17g00102*) showed similar tendency to *VvFLA7a*, except for up-regulated significantly in ‘Red Globe’ at maturation (Fig. 5l). Moreover, the expression of the candidate gene *VvNAC83* (*Vitvi17g00066*) showed no significant difference between ‘Muscat Hamburg’ and ‘Red Globe’ at preveraison but showed a higher expression level in ‘Muscat Hamburg’ than in ‘Red Globe’ at maturation, and the expression of *Vvβ-1,3-galactosyltransferase 2* (*Vitvi10g00092*) showed a tendency similar to that of *VvNAC83* between ‘Red Globe’ and ‘Muscat Hamburg’ during maturation (Fig. 5m and n).

### Correlation between VvWARK2 and berry texture

**Figure 6:**
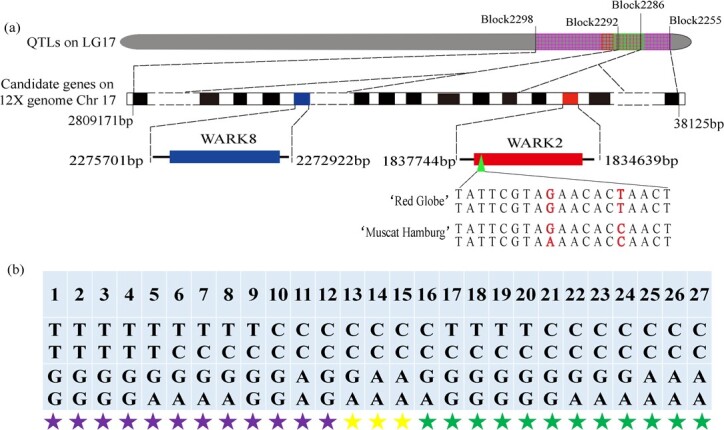
Schematic graph of *VvWARK2* and *VvWARK8* in the grape 12X.v2 genome and SNP effect analysis. (**a**) *VvWARK2* and *VvWARK8* refer to chromosome 17. (**b**) The ‘T/C’ (Chr17:1836764) and ‘G/A’ (Chr17:1836770) SNPs identified in 27 cultivars (lines). Purple, yellow, and green stars represent hard-, medium-, and soft-textured grape cultivars (lines), respectively. 1 to 27 represent the grape cultivars (lines) ‘Changezhi’, ‘Red Globe’, ‘Christmas Rose’, ‘Sweet Sapphire’, ‘Crimson Seedless’, ‘Thompson Seedless’, ‘Shennong Cuifeng’, ‘Tiangong Cuixiangmi’, ‘9–22’, ‘Blackcrunchy Seedless’, ‘Queen Nina’, ‘Cuiguang’, ‘Moldova’, ‘Zaoxia Meigui’, ‘Hongyanxiang’, ‘Muscat Hamburg’, ‘9–1-358’, ‘15–71’, ‘9–1-254’, ‘Zhuosexiang’, ‘Beibinghong’, ‘Takatsuma’, ‘Venus Seedless’, ‘Beta’, ‘Kyoho’, ‘Shennong Jinhuanghou’, and ‘Zui Jinxiang’, respectively.

Alpha diversity analysis was employed to analyse the relationships between berry texture values for detected stable QTLs and the genotypes of block markers located in the candidate genes. The marker Block2286 correlated significantly with MesF in 2018 and 2019 and PPH in 2019 and was found to be anchored to the promising candidate gene *VvWARK2* (Fig. S4, see online supplementary material), and two SNPs were detected according to the *VvWARK2* resequencing data from ‘Muscat Hamburg’ and ‘Red Globe’. The hard-textured grape ‘Red Globe’ carries homozygous alleles (TT/GG genotype) for *VvWARK2* at Chr17:1836764 and Chr17:1836770; however, the soft-textured grape ‘Muscat Hamburg’ carries the ‘CC’ and ‘GA’ genotypes at Chr17:1836764 and Chr17:1836770 (Fig. 6a). Additional grape germplasm sequencing revealed that *VvWARK2* carrying the ‘CC’ and ‘GA’ genotypes at Chr17:1836764 and Chr17:1836770 may be associated with non-hard texture grape cultivars (Fig. 6b).

## Discussion

### Complex grape berry texture with high heritability of traits

Berry texture is an important factor in table grapes and affects edible quality and shelf life after harvest [44]. Both the mesocarp (flesh) and pericarp (peel) of berries affect their texture. The mesocarp firmness of a grape berry can roughly be categorized as very firm, slightly firm, medium, or soft [15]. The high mesocarp firmness of a table grape shows the desirable brittleness texture and decreased loss during storage, which are the characteristics desired by most grape breeders at present. However, the pericarp thickness of berries also has a profound impact on the taste of grapes, and a thicker pericarp may render grape berries more elastic and less likely to crack [46]. In this study, the high-mesocarp firmness (MesF) grape ‘Red Globe’ showed higher PPH than the soft-mesocarp grape ‘Muscat Hamburg’ but lower PerB (Fig. 1). MesF, PPH, and PerB displayed continuous variation and normal distributions in the ‘Red Globe’ × ‘Muscat Hamburg’ (RM) population (Fig. 1), which is consistent with texture being a typical quantitative trait in grape berries [8–11, 14]. On the other hand, Correa *et al.* [9] reported that the broad-sense heritability (*H^2^*) of berry firmness (flesh and skin) was 0.86841 to 0.94843 and Ban et al. [8] reported that the *H^2^* of berry firmness was 0.813 based on sensory evaluation with each individual on a scale of 1–5. In the present study, the MesF, PPH, and PerB of berries were analysed individually and showed high *H^2^* in different years (Table 1), which is consistent with the findings of previous studies [8, 14]. However, correlation analysis of the RM population showed no significant correlation (*P* < 0.05) between MesF and PerB, even though PPH exhibited a significant correlation with both MesF and PerB (Fig. 2). This result was also supported by QTL identification. The QTLs for MesF did not overlap well with those for PerB, although in some QTL regions in LGs 6 and 14, the QTL for MesF was close to that for PerB. These results suggest that it is difficult to obtain high-MesF and high-PPH grape cultivars with high PerB.

### High-density genetic linkage map construction for grape

A reliable genetic map is essential for identifying QTLs and candidate genes for traits of interest [47]. Because grapes are highly heterozygous and have long breeding cycles, the F_1_ population is usually used as a mapping population based on the pseudo-testcross strategy, and QTL studies of grape traits based on lower marker numbers (<1000) with relatively broad QTL intervals may affect candidate gene identification [14, 48]. To improve the precision of QTL mapping and efficiency of candidate gene identification, more markers are usually used to increase the density of genetic maps or multiple omics methods, such as RNA-Seq, are combined [18, 11, 49]. In recent years, SNP markers obtained by next-generation sequencing (NGS) have been widely used to construct high-density genetic maps of grapes [50, 51] because SNP markers are widely distributed in genomic DNA sequences and possess better genetic stability, higher accuracy, and improved resolution [52, 53]. Specifically, according to the complete reference genome of the cultivar PN40024 (*V. vinifera* L.) assembled in a telomere-to-telomere (T2T) manner [54], NGS-based SNP genetic maps will be widely used for grape genetic trait analysis. In this study, whole-genome sequencing was applied to explore SNP molecular markers. The genetic map obtained contains 2725 bin markers (93 127 SNPs) with an average marker distance of 0.59 cM, which is smaller than the average marker distance (1.1–1.8 cM) in the genetic maps constructed in recent years [55–57]. Thus, the linkage map developed in this study is of high quality for further QTL identification and candidate gene analysis.

**Table 3:** Summary of QTLs for MesF, PPH, and PerB in the consensus map.

Trait	LG	QTL	Year	LOD threshold	LOD score	LOD peak (cM)	CI extremes (cM)	Flanking markers	R^2^
MesF	1	MesF1.1	2017	4.6	6.1	66.3	52.1–67.0	Block118-Block135	20.6
	4	MesF4.1	2017	4.7	4.4	49.3	48.4–50.4	Block3750-Block3754	14.3
	6	MesF6.1	2017	4.6	5.9	21.6	18.6–22.3	Block4266-Block4277	20.1
	8	MesF8.1	2019	4.7	4.7	88.3	86.7–91.6	Block4955-Block4969	5.7
	10	MesF10.1	2018	3.0	7.4	2.0	1.0–7.3	Block396-Block406	13.1
	10	MesF10.2	2019	3.1	14.2	4.3	2.6–4.9	Block400-Block404	20.7
	10	MesF10.3	2019	3.1	8.1	39.8	38.1–41.1	Block466-Block471	10.6
	11	MesF11.1	2018	3.7	6.8	75.1	70.5–79.5	Block744-Block727	11.9
	11	MesF11.2	2019	3.1	5.7	6.7	6.3–8.7	Block588-Block592	7.1
	11	MesF11.3	2019	3.1	6.5	69.2	68.5–69.9	Block740-Block743	8.2
	14	MesF14.1	2019	3.0	7.7	92.1	86.1–92.1	Block1782-Block1770	10.1
	17	MesF17.1	2018	3.0	8.1	66.0	62.8–68.1	Block2276-Block2263	16.1
	17	MesF17.2	2019	3.0	5.2	68.8	53.8–72.1	Block2298-Block2255	6.5
PPH	6	PPH6.1	2018	5.2	9.1	11.6	10.9–14.0.9	Block4249-Block4258	17.2
	10	PPH10.1	2019	3.1	4.7	20.0	14.5–22.5	Block420-Block424	9.0
	11	PPH11.1	2017	3.6	13.9	37.2	34.3–37.2	Block644-Block650	25.8
	11	PPH11.2	2018	3.6	4.9	77.0	75.0–85.0	Block725-Block738	8.8
	11	PPH11.3	2019	3.4	3.6	68.6	61.0–75.1	Block739-Block724	6.8
	14	PPH14.1	2018	3.8	5.5	89.0	86.4–92.0	Block1781-Block1770	9.9
	16	PPH16.1	2017	3.2	7.5	73.7	72.7–78.7	Block2124-Block2117	11.6
	16	PPH16.2	2019	3.1	4.7	88.4	86.1–92.7	Block2102-Block2087	9.1
	17	PPH17.1	2019	2.9	7.7	66.5	65.8–68.5	Block2267-Block2262	15.6
PerB	3	PerB3.1	2018	4.7	6.8	58.0	56.5–58.2	Block3574-Block3569	10.5
	6	PerB6.1	2018	4.7	9.1	6.7	4.6–7.3	Block4232-Block4239	14.4
	9	PerB9.1	2018	4.8	10.7	14.7	13.9–15.6	Block5156-Block5160	17.4
	14	PerB14.1	2018	3.0	9.4	81.8	74.1–86.7	Block1811-Block1780	15.1
	16	PerB16.1	2018	3.1	5.5	29.7	24.1–31.7	Block2209-Block2191	8.3

**Table 4:** Candidate genes potentially associated with berry texture with reliable QTLs identified.

Grapevine 12X.v2 gene ID	LG	Position in 12X.v2	Annotation or putative gene function	Reference
Vitvi10g00085	10	880 560–882 396	Glucan endo-1,3-β-glucosidase 1	[40]
Vitvi10g00092	10	964 308–971 029	β-1,3-galactosyltransferase 2	[41]
Vitvi11g00950	11	12 982 113–12 984 558	FLA 7	[42]
Vitvi11g00975	11	13 499 018–13 501 420	FLA 7a	[42]
Vitvi14g00996	14	18 305 259–18 308 930	β-glucosidase 44	[43, 44]
Vitvi17g00106	17	1 079 814–1 080 154	Glucuronoxylan 4-O-methyltransferase 1	[45]
Vitvi17g00175	17	1 836 665–1 837 771	WARK2	[44]
Vitvi17g00209	17	2 272 922–2 275 701	WARK8	[44]
Vitvi17g00234	17	2 627 614–2 630 248	β-glucosidase	
Vitvi10g00063	10	618 767–620 863	WRKY31	
Vitvi17g00025	17	240 653–242 053	ERF113	
Vitvi17g00066	17	639 252–640 234	NAC83	
Vitvi17g00102	17	1 054 544–056577	WRKY1	
Vitvi17g00231	17	2 589 991–2 591 000	MYB4-like	

### Reliable QTLs and candidate genes for berry texture

Previous studies have shown that berry texture is a typical quantitative trait controlled by multiple genes [9, 30]. To date, numerous QTLs for berry texture have been detected in LGs 1, 2, 3, 4, 5, 8, 9, 10, 13, 15, 16, 17 and 18 using different evaluation methods involving sensory evaluation, berry firmness (flesh and skin), and 20% deformation of berry height [8–11, 14–17]. However, there are no reports clearly distinguishing QTLs for MesF, PPH, and PerB. In this study, thirteen, nine, and five QTLs for MesF, PPH, and PerB, respectively, were independently identified in LGs 1, 3, 4, 6, 8, 9, 10, 11, 14, 16, and 17 (Fig. 4), and the QTLs for berry texture in LGs 6, 11, and 14 were detected for the first time. The results provide a specific basis for the directional improvement of grape texture.

For grape berry texture, a major QTL was identified in LG 18 in previous studies. Indeed, Carreño *et al.* [14] detected a major QTL for berry firmness in LG 18 near the SSR marker VMC6F11, explaining 19.8% of the total phenotypic variance. Correa *et al.* [9] found that the QTL for berry firmness was near the SSR marker VVIN16. Jiang *et al.* [11] found three QTLs for berry firmness named *qBF18–2016,* q*BF18–2017*, and q*BF18–2018* in LG 18, which corresponded to the grape genome region chr18: 24639353–28 587 457 and were related to the candidate genes *abscisic-aldehyde oxidase-like*, *endoglucanase 3*, and *NAC 90-like,* respectively. In addition, a major QTL for berry firmness reported by Crespan *et al.* [10] in LG 18 collocated with the SSR marker linked to *VviAGL11*, which is a main gene linked to seedlessness. In this study, major QTLs for berry texture were not identified in LG 18 by multiple QTL mapping; however, we did detect two QTLs for MesF in LG 18 by single-interval mapping in 2018 and 2019 (Table S9, see online supplementary material). These results indicate that the QTL for MesF in LG 18 detected in the study is not stable, and a similar result was found by Correa *et al.* [9]: the QTL detected in LG 18 was not as stable as the QTL in LG 8 in different seasons, and the QTL in LG 18 was localized to different positions in this LG in another study [14]. In this study, an unstable QTL (MesF8.1) was identified for MesF in LG 8, consistent with some major QTLs for berry firmness in LG 8 [9, 16, 18]. These results indicate that grape texture is greatly affected by genetic background.

In this study, a main QTL region (MesF17.1, MesF17.2, and PPH17.1) for MesF and PPH was detected in LG 17 between Block2298 (Chr17: 7998–532 287) and Block2255 (Chr17: 2805906–2 809 152) in 2018 and 2019, with good overlap, and explained up to 16.1% of the total phenotypic variance, with LOD peaks near markers Block2261, Block2264 and Block2265 (Chr17: 657561–706 586) (Table 3; Table S5, see online supplementary material). According to the physical location of QTL linkage markers in the reference genome, the QTLs MesF17.1, MesF17.2, and PPH17.1 are in the same region on chromosome 17, in which a QTL detected by Crespan *et al.* [10] was associated with SSR markers VMC3A9 (Chr17: 5115923–5 115 326) but in a different position in LG 17 (Chr17:14485590) compared with that of the QTL identified by Guo *et al.* [15]. In the stable QTL confidence interval, two wall-associated receptor kinase genes, *VvWARK2* and *VvWARK8*, are considered promising candidate genes because WARK is a transmembrane receptor that can participate in cell integrity maintenance [58] and directly combined with pectin residue in the cell wall [59]. The more important concern is that these two identical genes were key factors in high berry firmness according to transcriptome analysis in our other study. The transcripts of *VvWARK2* and *VvWARK8* were more highly expressed in the firm-fleshed cultivar ‘Red Globe’ than in the soft-fleshed cultivar ‘Muscat Hamburg’ from preveraison to maturation [44]. In this study, *VvWARK2* and *VvWARK8* were more highly expressed in ‘Red Globe’ than in the soft-fleshed cultivar ‘Muscat Hamburg’ during ripening (Fig. 5), which was in agreement with the results of our previous study. The high expression level of the *VvWARK8* gene in the firm-fleshed cultivar ‘Red Globe’ may increase pectin residue binding with WARK for high berry firmness maintenance according to weighted gene coexpression network analysis; however, a similar result was not found for the *VvWARK2* gene [44]. In this study, *VvWARK2* was found to include SNPs with the ‘CC’ and ‘GA’ genotypes at Chr17:1836764 and Chr17:1836770, which may be associated with non-hard texture grape cultivars (Fig. 6). The results showed that the molecular mechanisms of *VvWARK2* and *VvWARK8* involvement in grape berry texture may be different, and more studies are needed to validate their function.

Two new stable QTLs for berry texture in LGs 11 and 14 were first detected in grape, and three candidate genes (*VvFLA7*, *VvFLA7a*, and *Vvβ-glucosidase44*) are believed to be related to texture because *Fasciclin-like arabinogalactan protein* contributes to plant cell wall integrity and plant stem strength by cellulose deposition [42] and *β-Glucosidase* decreases fruit softening during ripening [43, 44]. In addition, Carreño *et al.* [14] identified a QTL for berry firmness in LG 10 near SSR marker VVIV37 (Chr10: 14591331–14 590 895) and Ban *et al.* [8] detected a QTL for berry firmness in LG 10 near SSR marker VVIH01 (Chr10: 1472920–1 473 487). In this study, a stable QTL for MesF (MesF10.1 and MesF10.2) was detected in LG 10 near bin marker Block406 (Chr10: 1217708–1 222 696) (Table 3; Table S5, see online supplementary material), which is in accord with results reported by Ban *et al.* [8]. The QTLs for berry texture detected in different LGs or different locations in one LG are in agreement with the observations that berry texture differences are probably due to linked genes and multiple genes with pleiotropic effects.

In this study, five TFs, including members of the WRKY, AP2/ERF, NAC, and MYB families, were identified in the stable QTL intervals of LGs 17 and 10. It is worth noting that these two stable QTLs on LGs 17 and 10 were also identified in previous studies [8, 10]. In recent years, studies have shown that AP2/ERF, WRKY, NAC, and MYB family TFs can regulate cell wall metabolism gene expression to influence fruit texture changes [36, 38, 60–63], and the expression of candidate genes for cell wall metabolism showed a trend similar to that of *VvMYB4-like*, *VvERF113*, *VvWRKY31*, *VvWRKY1*, and *VvNAC83* (Fig. 5). These results suggest that the promising candidate TF genes may regulate the expression of candidate genes for cell wall metabolism to influence grape berry texture changes.

## Materials and methods

### Plant material

An F_1_ population derived from the cross between ‘Red Globe’ (*V. vinifera* L.) and ‘Muscat Hamburg’ (*V. vinifera* L.) was used in the present study (*n* = 151). The female parent ‘Red Globe’ has a firm-flesh texture after maturation, and the male parent ‘Muscat Hamburg’ has a soft-flesh texture after maturation. Intraspecific hybridization was conducted in May 2011, and hybrid seeds were collected in October 2011. The offspring and parents were cultivated at Shenyang Agricultural University with commercial vineyard management and pruning. Genomic DNA (gDNA) was extracted from young leaves of the parents and offspring. Clusters were harvested from each plant at maturity from 2017 to 2019, 30 similarly sized berries were used for texture evaluation immediately, and 10 berries were frozen with liquid nitrogen for the following experiment after sampling.

### Grape berry texture determination

The berry mesocarp (flesh) firmness (MesF), pericarp (peel) puncture hardness (PPH), and pericarp brittleness (PerB) were determined using a texture analyzer (TA. XT Express, Stable Micro System, Godalming, UK) according to a previous method, with some modifications [64]. MesF indicated the average force (*g*) required to puncture the berry flesh, PPH indicated the force (*g*) used from the probe touching the peel until the peel is punctured and PerB indicated the displacement distance (mm) from the probe touching the peel to puncture peel. Berry puncture determination was performed at 1 mm/s until the depth reached 7 mm in the berry mesocarp using a needle probe with a diameter of 2 mm (P/2). Then, the probe was reset with a speed of 10 mm/s.

### Library construction for whole-genome resequencing

Total gDNA was extracted by using the CTAB method [65]. The high-quality gDNA of the two parents and 151 individuals was sheared into approximately 350 bp fragments by a Covaris ultrasonicator (S2/E210, Covaris Inc., Woburn, MA, USA). After end repair, barcodes and Illumina sequencing adapters were ligated to the single-nucleotide (A) repaired fragments using a previously described method [66]. Then, polymerase chain reaction (PCR) product purification and pooled sample separation were performed by using a previously described method [67]. Gel-purified products were used for paired-end sequencing (each end at 150 bp) by an Illumina HiSeq X Ten/NovaSeq system 6000 sequencer (Illumina, Inc., San Diego, CA, USA) according to a standard protocol.

### Marker development and genotype calling

To ensure that the reads were reliable, Illumina-sequenced raw reads for the genomic survey were first filtered using the fastp (v 0.20.1) preprocessor to remove the low-quality reads, including the number of base with quality value Q ≤ 10 accounts for more than 50% of the read (parameters: -q 10 -u 50), the average quality value of the read less than 20 (parameters: -e 20), the proportion of N (unable to determine the specific base type) on the read was greater than 10% (parameters: -y -Y 10), and the length of the paired-end reads was less than 100 bp or greater than 150 bp (parameters: -l 100 -b 150 -B 150), and then the Q30 and GC contents were calculated using an in-house Perl script. After excluding low-quality reads (Phred Q20, 99% certainty), raw reads from the two parents and each progeny were sorted according to their barcode sequences, and high-quality reads and clean reads were trimmed from the same sample and mapped to the grape reference genome (https://plants.ensembl.org/Vitis_vinifera/Info/Index) by Burrows-Wheeler Aligner (BWA) software [68]. Mark duplicates was executed by the Picard tool (http://sourceforge.net/projects/picard/) to reduce the impact of PCR duplication. To ensure the accuracy of the detected SNPs and InDels, the ‘Local realignment’ and ‘InDel realignment’ procedure were executed in the Genome Analysis Toolkit (GATK) v3.6 software using RealignerTargetCreator and IndelRealigner with default parameters. Afterward, the GATK’s HaplotypeCaller was used for SNP and InDel variant calling by generating the genomic VCF (GVCF) files, and the SNPs and InDels were strict filtered by the Bcftools and GATK, including SNPs at least 5 bp away from any InDels, SNPs separated by distances of more than 5 bp, and InDels separated by distances greater than 10 bp. Finally, the SNP set was generated by combining GATK and SAMtools with default parameters [69]. Then, the genotype of the F_1_ offspring was confirmed at a particular SNP locus. To merge the SNP dataset, the ‘pileup’ function in SAMtools was employed, and the genotypes of all SNPs were consistent at the loci in offspring and parents. SNPs identified as polymorphic markers should have two to four alleles between parents, and the polymorphic SNP markers were coded according to the cross-pollinated (CP) populations, which consisted of six segregation types (hk × hk, ab×cd, ef × eg, aa×bb, nn × np, and lm × ll).

### Bin marker calling and linkage map construction

SNP markers that showed less than 4× coverage in both parents and those from unknown chromosomes were discarded. A, SNP marker was retained every 2 kb to ensure a uniform distribution and a high density of SNPs. The chi-square (χ^2^) test was carried out for goodness-of-fit assessment at a confidence level of 0.01, and the SNP markers showing extreme segregation distortion (P < 0.01) were excluded from linkage map construction.

LGs were constructed using a previously described method [70], with some modifications. In brief, recombinant frequencies were calculated by two-point test crosses, and error genotype correction and missing genotype filling were conducted based on these linkage phases. Genotypes were called according to SNP ratios and remained unchanged until reaching the recombination breakpoint. This recombination breakpoint was defined as the boundary between heterozygous and homozygous genotypes, bin markers were generated by combining the SNP markers in the interval between each pair of consecutive breakpoints [71], and the linkage map was constructed using HighMap according to the recombination bin markers.

### QTL mapping and candidate gene analysis

QTL identification was performed according to previously described methods [72, 73]. QTL mapping was carried out on the consensus map using the berry texture traits (MesF, PPH, and PerB) evaluated in each year (2017, 2018, and 2019). The data loading map, phenotype, and genotype files in MapQTL format were processed using the R/qtl package [74] with a four-way cross format. Single-interval mapping was carried out with the ‘scanone’ function, and the ‘stepwiseqtl’ function and ‘hk’ option were used for multiple QTL mapping after single-interval mapping. The maximum QTL number (max.qtl) was set to two more than the QTL number detected with single-interval mapping, which is favorable for backward/forward selection. The genome-wide LOD threshold (α =0.05) was calculated by 1000 permutation tests, and the 95% QTL location confidence interval (CI) was derived using the ‘bayesint’ function (prob = 0.95). A QTL for berry texture was considered stable if it was identified for more than 2 years or more than two traits in the same LG region. QTL confidence intervals in the LGs were illustrated using MapChart 2.2 [75].

The physical interval limits were determined by the flanking bin markers of the CI for stable QTLs, and the candidate genes were identified according to the CI of each stable QTL on the consensus map [76, 77]. Candidate genes for berry texture were defined as promising candidate genes based on their potential functional annotations and the available literature. Candidate genes and their functional annotations were obtained from the 12X.v2 grapevine genome in the URGI database, and the candidate genes were selected based on the position of bin markers on the physical map. Promising candidate genes were identified for further investigation based on potential gene functions that may cause texture changes and the literature. The special primers used for *VvWARK2* SNP examination in grape germplasm are listed in Table S10 (see online supplementary material).

### Pedigree analysis

An in-house Perl script was used to screen the diverse InDels between ‘Red Globe’ and ‘Muscat Hamburg’, and 40–45 bp InDels were specifically isolated. According to the physical location of InDels in the grape genome, fifty-four 40–45 bp InDels within the coding area were randomly selected from the 19 chromosomes, and the forward and reverse primers located within 150 up upstream and downstream of InDels were designed for pedigree analysis of the parents (Table S11, see online supplementary material). In addition, the SNPs within the promising candidate gene area and 2000 bp upstream of the starting codon (ATG) were screened for KSAP analysis, and five KSAP primers were successfully designed for pedigree analysis (Table S12, see online supplementary material). The pedigree was constructed using R software with the neighbor-joining method and the ‘ape’ package.

### RT–qPCR validation

Ten promising candidates for berry texture were selected for RT–qPCR verification. RT–qPCR quantification was performed with an ABI QuantStudio 6 Flex System (Applied Biosystems, Foster City, CA, USA). Total RNA was extracted from ‘Muscat Hamburg’ and ‘Red Globe’ berry skins and mesocarps according to a previous report [78], with some modifications. The special RT–qPCR primers used in this study are listed in Table S13 (see online supplementary material). RT–qPCR was performed as previously described [73], and the expression level was calculated as 2^-ΔΔCt^ according to the Ct value of *VvActin* [78].

### Data analysis

The correlations between MesF, PPH, and PerB within different years were analysed by the nonparametric Spearman correlation coefficient in the R 4.1.2 package (R Development Core Team 2014). The distribution of MesF, PPH, and PerB in the F_1_ population was assessed using the Shapiro–Wilk test. The broad-sense heritability (*H^2^*) was calculated based on a previous report [79]. Candidate gene expression was analysed as the average ± standard error of three replicates using one-way ANOVA and Tukey’s test (P ≤ 0.05). The effects of SNP markers on berry texture were determined using the Kruskal–Wallis test and visualized using ImageGP [80].

## Supplementary Material

Web_Material_uhad226

## Data Availability

The data that support the results are included in this article and supplementary materials. The whole-genome resequencing data reported in this paper have been deposited in the NCBI BioProject under accession number PRJNA1018136.
